# Chimeric antigen receptor-macrophages: Emerging next-generation cell therapy for brain cancer

**DOI:** 10.1093/noajnl/vdaf059

**Published:** 2025-03-19

**Authors:** Myrthe J A Koppers, Matthijs Monnikhof, Jan Meeldijk, Thijs Koorman, Niels Bovenschen

**Affiliations:** Department of Pathology, University Medical Center Utrecht, Utrecht, The Netherlands; Department of Pathology, University Medical Center Utrecht, Utrecht, The Netherlands; Center for Translational Immunology, University Medical Center Utrecht, Utrecht, The Netherlands; Department of Pathology, University Medical Center Utrecht, Utrecht, The Netherlands; Department of Pathology, University Medical Center Utrecht, Utrecht, The Netherlands; Center for Translational Immunology, University Medical Center Utrecht, Utrecht, The Netherlands; Department of Pathology, University Medical Center Utrecht, Utrecht, The Netherlands

**Keywords:** adoptive cell therapy, brain tumor, chimeric antigen receptor, CAR-macrophages, tumor microenvironment

## Abstract

Adoptive cell-based therapy utilizing chimeric antigen receptor (CAR)-T technology holds promise in the field of neuro-oncology. Significant progress has been made in enhancing both the efficacy and safety of CAR-T-cell therapies. However, challenges such as the multifaceted immunosuppressive impact of the tumor microenvironment and insufficient CAR-T-cell infiltration into brain tumor sites remain a major hurdles. Emerging novel approaches utilizing CAR-macrophages (CAR-MACs) show potent results for brain tumor immunotherapy. CAR-MACs localize to tumor sites more readily, increase immune cell infiltrates, and demonstrate high antitumor efficacy by effectively eliminating tumor cells through mechanisms such as phagocytosis or efferocytosis. This review discusses the current advancements in CAR-MAC cell therapies for brain cancer, followed by an overview of research on manufacturing CAR-MACs for clinical application. We further highlight the potential future applications of CAR-MACs in combinatory therapies in the treatment of brain tumors.

## Graphical Abstract

**Graphical Abstract:**
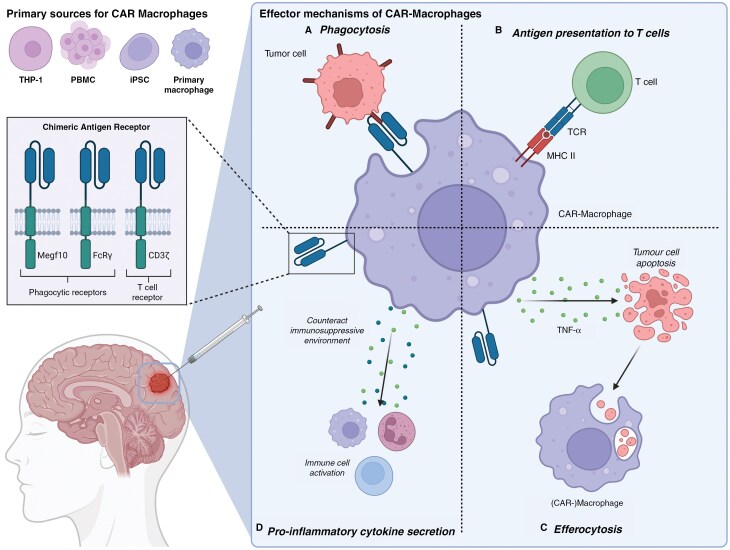

**Key Points**

- CAR-Macrophages are characterized by their high tumor infiltration, low toxicity, and high cytotoxicity against brain tumors.
- CAR-Macrophages can reverse the immunosuppressive brain tumor microenvironment.
- Large-scale CAR-Macrophage production can be manufactured ‘off-the-shelf’ and in vivo.

Primary brain tumors originate from central nervous system cells and are destructive types of cancer. Over 75% of adult brain tumors are diagnosed as gliomas, compared to the less common, for example, medulloblastomas and ependymomas.^1^ Glioblastomas represent the most aggressive form in adults, having a median survival of less than 1 year.^2^ Glioblastoma is also one of the most lethal solid brain cancers in pediatric patients, though they are less common than other high-grade tumors like diffuse midline gliomas.^3^ Both types pose significant risks to children’s health and treating brain tumors is particularly challenging due to their intricate biological characteristics.^4^ Brain tumors often have unique genetic and molecular profiles that complicate diagnosis and hinder effective treatment.^5^ These complexities highlight the urgent need for innovative strategies to improve outcomes for patients. Standard of care is limited to surgery, radiation and chemotherapy, and few effective treatments remain after recurrence.^6^ Immunotherapy is a promising example of future treatment currently being investigated.^7,8^

Adoptive cellular immunotherapies are revolutionary in the treatment of hematological cancer, but obstacles such as tumor heterogeneity and the microenvironment remain challenging to play an effective role in solid tumors.^9^ Chimeric antigen receptor (CAR) therapies show promising results in brain tumors, for example, 50% of patients with recurrent high-grade glioma achieve stable disease or better following treatment with IL-13Rα2 targeted CAR-T-cells.^10^ However, the hurdles related to CAR-T-cell therapies are similar to other solid tumors. Macrophages (MACs) are highly effective in tumor infiltration and influencing the tumor microenvironment (TME).^11^ As a novel approach, CAR-macrophages (CAR-MACs) gain interest and show promising results in solid tumors, potentially overcoming challenges like the immunosuppressive TME that limits CAR-T-cell efficacy.

In the present review, we discuss the advantages and disadvantages of CAR-T-cell therapy for brain tumor treatment and examine the current advancements in CAR-MAC cell therapies for brain cancer. Additionally, we provide an overview of research on manufacturing CAR-MACs for clinical application and highlight the potential future applications of CAR-MACs in combinatory therapies in the treatment of brain tumors.

## CAR Therapy

CAR therapy has revolutionized cancer treatment.^9^ Adoptive cell therapy aims to boost and target the natural anti-cancer activity of multiple types of immune cells through ex vivo activation and the introduction of engineered receptors that recognize tumor-specific (neo)antigens.^9^ As such, CAR therapy is both a powerful alternative or adjunctive therapy to existing approaches, such as surgery, radiation therapy, and chemotherapy. Genetically engineered T-cells are predominantly utilized in CAR therapy and the CAR surface proteins mediate major histocompatibility complex-independent tumor cell lysis.^12^ A CAR molecule is comprised of an antigen-binding domain specifically directed towards a tumor-associated antigen, a hinge region to modulate the length of the extracellular segment, a transmembrane domain for embedding the CAR in the membrane, connected to intracellular (eg, T-cell specific) cell activating and co-stimulatory signaling domains.^9^ Upon antigen binding, the downstream signaling cascade is triggered, which activates the immune cell to engage in tumor-specific cell lysis. Currently, multiple clinical trials of CAR-T and CAR-Natural Killer cells are ongoing for brain tumors, with CAR-T being the most abundant.^13^

### CAR-T-Cell Therapy in Brain Tumors

**Figure 1.**
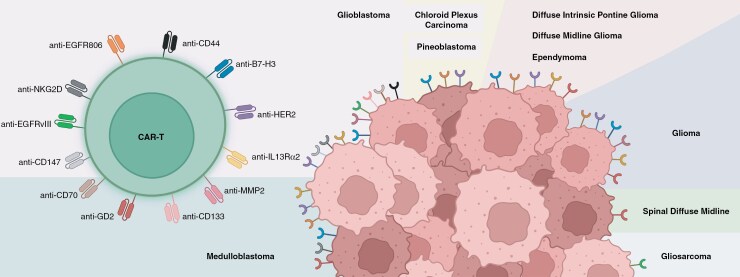
Schematic overview of chimeric antigen receptor (CAR)-T-cell therapies currently undergoing clinical trials for brain tumor treatment. Tumor cells are depicted with distinct antigens, each color-coded to correspond with their respective targeting CAR on the T-cell. This color alignment illustrates the specificity of the CAR-T-cells for the antigens expressed across different tumor types in clinical trials.

Several CAR-T-cell therapies are approved by the United States Food and Drug Administration (FDA) to treat patients suffering from hematological malignancies.^14^ Therapies targeting solid tumors (including brain tumors) have not yet received FDA approval and are still in the clinical trial phase.^15^ Despite potential complications, numerous clinical trials are currently investigating CAR-T-cell therapies targeting brain tumors through various antigens, such as EGFR, IL13Rα2, B7-H3, GD2, and CD70 (Figure 1, Table S1). Preliminary data indicate that CAR-T-cells can successfully cross the blood-brain barrier (BBB) and localize to tumor sites.^16^ However, safety concerns remain, as patients receiving higher CAR-T-cell doses experience adverse events.^10,16–18^

**Figure 2.**
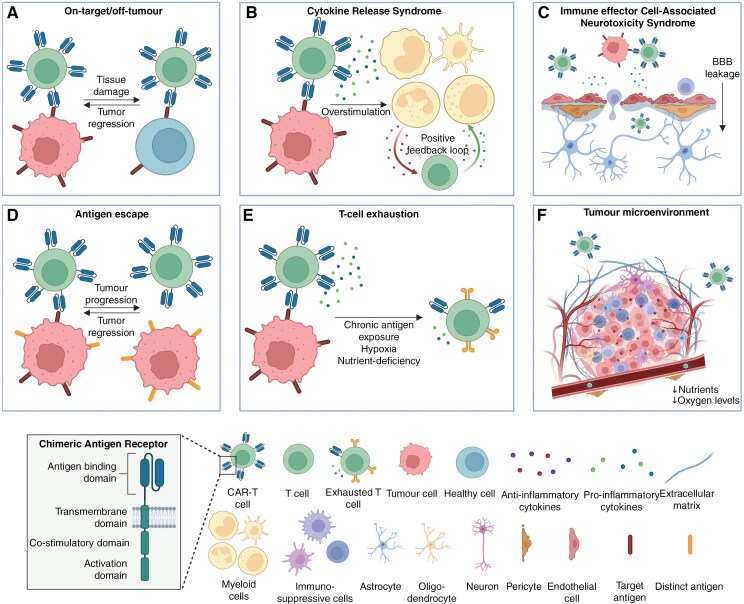
Limitations of chimeric antigen receptor (CAR)-T-cell therapy. Safety and efficacy challenges in CAR-T-cell therapy include (A) on-target/off-tumor cytotoxicity, (B) cytokine release syndrome and (C) immune effector cell-associated neurotoxicity syndrome. The efficacy is limited due to (D) antigen escape, (E) CAR-T-cell exhaustion and (F) the brain tumor microenvironment.

A systematic review including 8 studies identified significant safety challenges in several Phase I trials for glioblastoma, where serious adverse events such as the on-target/off-tumor effect, cytokine release syndrome (CRS), and immune effector cell-associated neurotoxicity syndrome (ICANS) were frequently observed (Figure 2, A–C).^19^ CRS and ICANS result from excessive cytokine secretion by CAR-T and activated surrounding immune cells, creating a disruption of BBB, allowing immune cells to infiltrate the brain and cause neurotoxicity and other life-threatening symptoms.^20–22^ Additionally, on-target/off-tumor toxicity can occur when a CAR is directed against an antigen expressed both on cancer cells and healthy tissue, which often occurs due to a lack of tumor-specific (neo)antigens.^20^ Another obstacle is the reactivation of latent human herpesvirus 6 during CAR-T-cell manufacturing can lead to severe toxic consequences.^23^ Moreover, a recently observed phenomenon described as localized tumor inflammation-associated neurotoxicity (TIAN) can arise following CAR-T-cell treatment and lead to local neurological deficits due to inflammation.^24^ These complications have raised considerable concerns regarding the overall risk profile of CAR-T-cell therapy in solid (brain) tumor treatment.

The efficacy of CAR-T treatment is challenged by the heterogeneity of brain tumors, along with CAR-T-cell exhaustion and immunosuppressive TME (Figure 2, D–F).^25,26^ Tumor heterogeneity enables antigen escape against CAR-T-cells targeting one single antigen, as tumor cells downregulate or alter antigen expression to evade recognition.^26^ In a clinical trial for recurrent glioblastoma (NCT02209376), 5 out of 7 patients showed reduced expression levels of target antigen EGFRvIII after CAR-T-cell infusion compared to pre-infusion, highlighting this challenge.^27^ The “escaped” cells can then divide and often expand, skewing mutational drift within cancer cell types, further complicating treatment efforts.^27^ Additionally, CAR-T-cells may experience exhaustion due to prolonged cytotoxic activation, mitochondrial dysfunction induced by a hypoxic environment or immunosuppressive signals in the TME, reducing their therapeutic efficacy.^25,28,29^ The TME is a physical and molecular barrier that comprises a complex immunosuppressive environment. It is composed of a specific matrix, enriched with hyaluronic acid and lower levels of collagens and laminins, along with tumor and immune cells, with MACs being the predominant immune cell population in glioblastoma.^30–33^ These cells secrete cytokines that inhibit immune responses, promoting tumor growth and CAR-T-cell impedance.^25^ Additionally, the TME of brain tumors is enriched with neurons and oligodendrocytes, which also support tumor progression.^34^ Pericytes and astrocytes further strengthen the BBB by encircling endothelial cells. This interaction creates an extra physical and chemical barrier, that restricts mainly adaptive immune cells and cytokine infiltration into the brain tumor.^35^

In recent years, extensive research focused on the TME to enhance the efficacy of CAR therapy for brain tumors, aiming to ensure that CAR-T-cells remain or become active specifically within the tumor. Various strategies have arisen to achieve this goal, including abating the immunosuppressive TME and developing CAR-T-cells that are more resilient to these immunosuppressive features. These approaches focus on augmenting pro-inflammatory cytokine secretion or exploiting the immunosuppressive characteristics of the TME as “on-switches” for CAR-T-cells. These strategies encompass metabolic engineering of CAR-T-cells, designing inverse signaling CAR-T-cells, and an adjuvant therapy approach with immune-cytokines.^36–38^ While these methods have not yet been explored for CAR-MACs, they hold the potential for improving CAR-MAC efficacy by leveraging similar immune-modulating mechanisms.

#### Metabolic engineered CAR-T cells

Hallmark features of the TME of brain tumors are hypoxia and nutrient scarcity.^39^ The enhanced metabolic activity of tumor cells leads to excessive consumption of oxygen and nutrients. The production of high amounts of secondary metabolites creates an environment that undermines anti-cancer immunity.^40,41^ These conditions pose challenges to the efficacy of CAR-T-cell therapy. Immune cells can be significantly inhibited by a shortage of primary nutrients and “metabolic poisoning” by secondary metabolites like adenosines.^42^ Consequently, T-cells get exhausted and become inactive, while MACs transition towards an anti-inflammatory phenotype.^43,44^ Although reactivating exhausted T-cells is extremely challenging, fortunately, MACs exhibit plasticity, allowing them to revert to an antitumor phenotype in response to (external) stimuli.^45,46^ To potentiate activity within the TME, CAR-T-cells have been engineered to maintain effector functions under hypoxic and/or nutrient-deprived conditions. Fernandez-Marcos et al. modified CAR-T cells with PPARγ coactivator 1α (PGC-1α) to enhance mitochondrial health and support oxidative phosphorylation and fatty acid oxidation.^47^ A recent study demonstrated that an anti-EGFR CAR-T-cell modified with an engineered version of PGC-1α shows improved mitochondrial biogenesis and enhanced antitumor activity in solid tumors.^36^ However, 40% of these CAR-T cells still exhibited an exhaustion phenotype marked by the expression of co-inhibitory receptors PD-1 and Tim-3, likely due to prolonged antigen exposure or antigen-independent activation through CAR clustering.^25,48^ A recent in vivo study by Hatae et al. showed that metabolic reprogramming of CAR-T-cells enhances mitochondrial biogenesis and improves their function in the hypoxic brain TME, resulting in better tumor control and increased resistance to immune exhaustion.^49^ This metabolic reprogramming approach could be extended to CAR-MACs. While PGC-1α upregulation enhances CAR-T-cell function, it drives MACs toward an anti-inflammatory state.^50^ Therefore, downregulation of PGC-1α could be beneficial for enhancing CAR-MAC activity.

An innovative strategy takes advantage of the hypoxic TME. The safety of CAR-T cells can be enhanced by incorporating oxygen-sensitive “on-switches” that activate in response to low oxygen levels. A characteristic feature of a hypoxic environment is the accumulation of hypoxia-inducible factor 1α (HIF-1α). Under normoxic conditions, HIF-1α is degraded following hydroxylation and ubiquitination.^51,52^ Juillerat et al. engineered a CAR with the oxygen-sensitive degradation domain (ODD) of HIF-1α integrated as an oxygen sensor.^52^ Optimization of this CAR system has led to the development of a HypoxiCAR.^53^ The HypoxiCAR features a dual oxygen-sensing mechanism, comprised of HIF-1a’s ODD and a sequence of 9 hypoxia-response elements (HRE) located in the long terminal repeat enhancer region of the CAR vector. In CAR-T cells in the TME, the undegraded HIF-1α due to low oxygen levels is harnessed as a transcription factor to promote CAR expression through binding to the HRE. HypoxiCAR-T-cells located at the tumor site in mice, effectively inhibit tumor growth without evident toxicity and safety issues.^53^ This HypoxiCAR-T-cell did not display significant antitumor activity under normoxic conditions in vitro.^53^ This highlights the enhanced safety profile of this CAR therapy, which could also be adapted for CAR-MACs to reduce the risk of inadvertently targeting healthy tissue in normoxic environments, thereby minimizing the on-target/off-tumor cytotoxicity.

#### Inverted cytokine receptor CAR-T-cells

The brain TME harbors numerous anti-inflammatory cytokines and stimulatory ligands secreted by tumor cells and surrounding immune cells, such as IL-4, IL-10, and TGF-β.^54^ These molecules diminish antitumor activity of infiltrating immune cells and thus enable tumor progression. To enhance the efficacy of CAR-T-cell therapy, extensive research has focused on utilizing anti-inflammatory cytokines as a stimulus to mitigate the immunosuppressive nature of the TME, resulting in the development of inverted cytokine receptors (ICRs). These ICRs generate a pro-inflammatory signal upon binding to an anti-inflammatory cytokine, thereby alleviating the immunosuppressive TME.^37,55,56^ Recently, an IL-4/IL-15 ICR, consisting of an IL-4 receptor extracellular domain and an IL-15 receptor transmembrane domain and endodomain, in combination with an NKG2D-CAR demonstrated improved antitumor activity of the CAR-T cells against pancreatic cancer in vivo.^55^ The IL-4/IL-15 CAR showed reduced T-cell exhaustion in the TME, increasing the CAR-T-cell efficacy. Further investigation is required to increase the specificity of brain tumors and to facilitate their application in CAR-MAC therapy. By engineering MACs to secrete pro-inflammatory cytokines, such as IL-12, IFN-γ, or TNF-α upon recognizing anti-inflammatory signals like IL-4, IL-10, or TGF-β, their polarization toward an antitumor phenotype can be promoted, potentially improving therapeutic outcomes in brain malignancies.^57^ However, the primary risk associated with ICRs is the potential for excessive immune activation, which can lead to severe toxicity and adverse side effects, such as autoimmunity and inflammatory damage to healthy tissues.

#### Combinatory approach with immune-cytokines

The immunosuppressive environment of solid tumors can be treated with pro-inflammatory cytokines to enhance immune cell activity and induce antitumor responses.^58^ However, many pro-inflammatory cytokines have a short half-life and pose significant toxicity risks, limiting their use in clinical applications.^59^ One way to harness pro-inflammatory cytokines in clinical settings is through immune cytokines. Immune-cytokines are fusion proteins comprising an antibody or single chain variable fragment (scFv) specifically directed towards a tumor-associated antigen, a linker, and a cytokine.^60^ Cytokines used in this approach could have 2 different goals: (1) inhibit tumor proliferation and stimulate cell death or (2) activate immune cells for an enhanced immune response. The promise of immune-cytokines in solid cancer treatment is demonstrated with 2 pleiotropic pro-inflammatory cytokine IL-12 molecules fused to an IgG1 antibody NHS76 (NHS-muIL12).^38^ In this study, treatment of mice with this NHS-muIL12 led to elevated serum levels of IFN-γ and triggered the activation of neighboring immune cells, inducing a pro-inflammatory response. A phase 1 clinical trial involving human NHS-IL12 (NCT01417546) has demonstrated that this immune-cytokine is well-tolerated in patients with metastatic solid tumors and effectively stimulates an antitumor response.^61^ Optimization of immune-cytokines has resulted in improved in vivo localization at the sites of solid tumors and enhanced antitumor responses.^62,63^ Given their capacity to augment antitumor responses, immune-cytokines are scrutinized in combination studies with CAR-T-cell therapy. Treatment with an anti-CEA-IL2 immune-cytokine in combination with anti-CEA CAR-T-cells directed against carcinoembryonic antigen (CEA) enhanced the efficacy of the CAR-T-cell therapy and resulted in tumor regression, demonstrating the added value of this combinatory approach for solid tumors.^64^ Additionally, this immune-cytokine could complement CAR-MAC therapy, as IL2 promotes macrophage polarization toward an antitumor phenotype, potentially expanding its therapeutic impact.^65^ Furthermore, this combination therapy may not only enhance CAR-MAC function but also stimulate an antitumor response in surrounding immune cells and reduce the exhaustion of infiltrating T cells.

## CAR-MACs as an Alternative Therapeutic Approach

It was commonly believed that innate immune cells exert only indirect effects on the TME by recruiting adaptive immune cells. This belief drove the development of therapies targeting adaptive immune cells, such as CAR-T-cell therapy. However, with a better understanding of the TME, it has become evident that innate immune cells play a crucial role in shaping the microenvironment.^30^ Consequently, innate immune cells have emerged as a focus of increasing interest for novel therapeutic strategies, including CAR-MAC therapy.

### MACs in the TME

MACs, one of the most abundant immune cells infiltrating brain tumors, are known as tumor-associated MACs (TAMs).^66^ TAMs are traditionally classified into 2 main phenotypes: M1 and M2.^67^ M1 MACs exhibit a pro-inflammatory phenotype, characterized by the expression of CD80, and CD86, whereas M2 MACs display a pro-tumor phenotype, marked by the expression of CD163 and CD206.^68^ Most brain tumors exhibit a high number of M2 TAMs, which either originate as M2 or transition from M1 MACs, to defend themselves against an immune response, by facilitating processes such as metastasis and angiogenesis.^31,68^ The immunosuppressive environment caused by the M2 TAMs can be dampened by M1 MACs in the TME.^67^ M1 MACs secrete pro-inflammatory cytokines to stimulate an immune response and recruit and activate T cells after antigen presentation, reshaping the TME.^66,69^ Additionally, M1 MACs can engulf tumor cells via phagocytosis.^70^ Although this research defined readout as M1 and M2, recent evidence reveals that TAMs exist along a polarization continuum, with MACs capable of reversible transitions between states with overlapping and somethings contradictory effector mechanisms. M1 MACs can inhibit neighboring M1 cells through cell density without transcriptomic differences, while M2 MACs also possess phagocytic capabilities. Beyond the traditional M1-M2 classification, TAMs are further classified into subgroups, for example, *HES1* TAMs and *TREM2* TAMs, based on Single-Cell Regulatory Network Inference and Clustering analysis.^71^ TAMs can also be divided into subgroups based on enriched pathways and predicted functions, for example, Inflam-TAMs and Angio-TAMs.^72^ While the M1-M2 paradigm is now considered oversimplified, these markers remain useful due to their prognostic significance in tumor models and human cancers. Although many immune cells such as T-cells encounter challenges in accessing brain tumors, MACs exhibit a remarkable ability to infiltrate and reside in the TME.^11,73^ Hence, MACs emerge as a promising strategy for antitumor therapies aiming to counteract the inhibitory effects of the TME to the current CAR-T-cell therapies. By incorporating CARs into MACs, these cells can be engineered to enhance their phagocytic capacity, allowing them to engulf and eliminate tumor cells more effectively.^74^ This does not only boost their ability to clear tumors expressing the targeted antigen, but it also improves their overall antitumor activity. MACs retain the ability to phagocytose tumor cells that evade detection by the CAR, avoiding antigen escape, and leading to better therapeutic outcomes in solid tumors including brain tumors.

### CAR-MAC milestones

CAR-MAC therapy represents an adaptation of CAR therapy, in which MACs are engineered to express a CAR to bind tumor cells. The first attempt to create CAR-MACs was a monocyte containing an anti-CEA scFv, a human Fc region hinge, and a transmembrane and intracellular domain of CD64, a receptor capable of facilitating tumor cell killing by MACs.^75^ The CAR constructs were transduced into monocytes using an adenoviral vector, resulting in the generation of CAR monocytes with antigen-specific cytokine secretion. However, antigen-dependent tumor cell phagocytosis was not observed because of the lack of specific intracellular activation domains.^75^ The discovery of the first CARs capable of triggering antigen-dependent tumor cell phagocytosis, encoding intracellular signaling domains Megf10, FcRγ, or CD3ζ, marked a significant breakthrough that propelled further research into CAR-MACs.^76^ CAR-MACs with intracellular domains of the phagocytic receptors Megf10 or FcRγ showed increased antigen-dependent phagocytosis compared to CAR-MACs with other intracellular domains.^76^ Moreover, CAR-MACs featuring a CD3ζ intracellular domain induced phagocytosis to a similar extent as CAR-MACs with intracellular domains of phagocytic receptors. This observation is remarkable, considering that CD3ζ is a T-cell receptor that binds ZAP70, a kinase that is not expressed in MACs. This study illustrated that CD3ζ within MACs interacts with Syk kinase, a signaling protein involved in phagocytosis, thereby promoting engulfment. Since the characterization of these phagocytosis-promoting intracellular domains, researchers have devised a variety of CAR structures tailored specifically for the modification of MACs, derived from various sources. Additional in vitro experiments showed that antigen presentation of phagocytosed tumor cells led to T-cell activation, thereby improving the antitumor response.^69^

The first in vivo findings originate from a study utilizing first-generation CAR-MACs with the CD3ζ intracellular domain.^69^ In this study, anti-CD19 CAR molecules targeting solid tumor antigens HER2 or mesothelin were transduced into THP-1 cell line MACs via adenoviral transfer, resulting in the generation of antitumor CAR-MACs. Across both a breast cancer and an ovarian cancer xenograft mouse model, CAR-MACs demonstrated infiltration into the TME, where they elicited an antigen-dependent antitumor response, including engulfment of tumor cells. Subsequent experiments using humanized immune system mouse models revealed that the CAR-MACs could reshape the TME towards a pro-inflammatory environment and activate surrounding immune cells upon antigen binding. The overall survival of mice was prolonged by 40% following intravenous injection and significantly enhanced after intraperitoneal injection.

These findings have prompted the initiation of multiple in vivo studies investigating CAR-MAC treatment for solid tumors (Table S2) and 3 studies specific to brain tumors (Table 1), which are discussed in more detail in the following chapters.^77–79^ While M1-associated intracellular domains from toll-like receptors (TLRs) and interferon-gamma receptors (IFNGRs) are under investigation for solid tumor treatment, CD3ζ remains the dominant choice for CAR-MACs targeting brain tumors.^69,80^ Notably, the MerTK intracellular domain has demonstrated superior phagocytosis and specific lysis compared to CD3ζ and TLRs in vitro.^81^ Its efficacy was further validated in vivo in a subcutaneous 4T1 breast cancer mouse model. However, this study lacks direct comparisons with other intracellular domains in vivo. To date, CD3ζ remains the preferred option for brain tumors due to its similarity to FcRγ, which mediates antibody-dependent phagocytosis effectively, and its extensive validation in CAR-T therapies, ensuring reliability in adapting CAR technology for MACs.^69^ Expanding research into alternative intracellular domains could enhance therapeutic options for brain tumor treatment by boosting either CAR-MAC phagocytic activity or M1 polarization.

**Table 1.** In Vivo CAR-Macrophage Studies Targeting Brain Tumors

Target	Activation domain	Cell source	In vivo	Delivery route	Primary endpoint	Reference
EGFRvIII	CD3ζ-TIR	Human induced pluripotent stem cells	Xenograft model of U87MG glioblastoma cells in NOD-SCID mice	Intraperitoneal; Intracraneal	Tumor volume, survival	Lei et al.^77^
CD133	CD3ζ	Primary macrophages	Syngeneic mouse model of C57BL/6 mice via orthotopic transplantation of GL261 glioma cells; Xenograft model of patient-derived glioblastoma cells in NOG-EXL mice via orthotopic transplantation	Intracranial; Intratumor	Survival, tumor burden	Chen et al.^78^
ERBB2/HER2	CD3ζ	Primary macrophages	Syngeneic mouse model of C57BL/6 mice via orthotopic transplantation of GL261 glioma cells; Xenograft model of patient-derived glioblastoma cells in huHSC-NOG-EXL mice via orthotopic intracranial injection	Intratumor	Tumor volume, mouse body weight, survival, tumor burden	Gao et al.^79^

EGFRvIII, epidermal growth factor receptor III; CD133, cluster of differentiation 133; ERBB2/HER2, human epithelial growth factor receptor 2.

Additionally, the first in vivo findings from Klichinsky et al. prompted the initiation of several clinical trials for solid tumors, directed against MSLN, HER2, or CD5 (Table 2). Given the safety concerns associated with CAR-T-cell therapy, the primary objective of these CAR-MAC trials is to assess safety and tolerability by evaluating the frequency and severity of adverse events. Unlike CAR-T-cells, which rely strictly on target antigen recognition for cytotoxic activity, CAR-MACs may be less affected by antigen escape. MACs can eliminate tumor cells through alternative mechanisms, such as “eat-me signals,” allowing them to continue their antitumor activity even when the target antigen is absent. This potentially leads to more effective tumor clearance.

**Table 2.** CAR-Macrophage Clinical Trials Targeting Solid Tumors

Clinical trial	Phase	Status	Tumor	Target	Delivery route	Estimated enrollment	Primary endpoint	Estimated primary completion date
NCT03608618	I	Terminated	Peritoneal Mesothelioma, Fallopian Tube Adenocarcinoma, and 2 more conditions	MSLN	i.p.	14	Treatment related AEs	02-2021
NCT04660929	I	Active, not recruiting	HER2-positive, adenocarcinoma and 29 more conditions	HER2	i.v. i.p.	48	Treatment related AEs and establish feasibility of manufacturing	12-2024
NCT05138458	I/II	Suspended	Peripheral T-cell lymphoma and cutaneous t-cell lymphoma	CD5	i.v.	40	Safety and tolerability	11-2024
NCT06224738	Early I	Not yet recruiting	Gastric cancer	HER2	i.p.	9	Treatment related AEs	03-2025
NCT06562647	Not Applicable	Recruiting	Ovarian Cancer	MSLN	i.v.	2	Maximum tolerated dose	04-2025

MSLN, mesothelin; HER2, human epithelial growth factor receptor 2; CD5, cluster of differentiation 5; AE, adverse events.

### CAR-MACs With Enhanced Polarization Towards the M1 Phenotype

**Figure 3.**
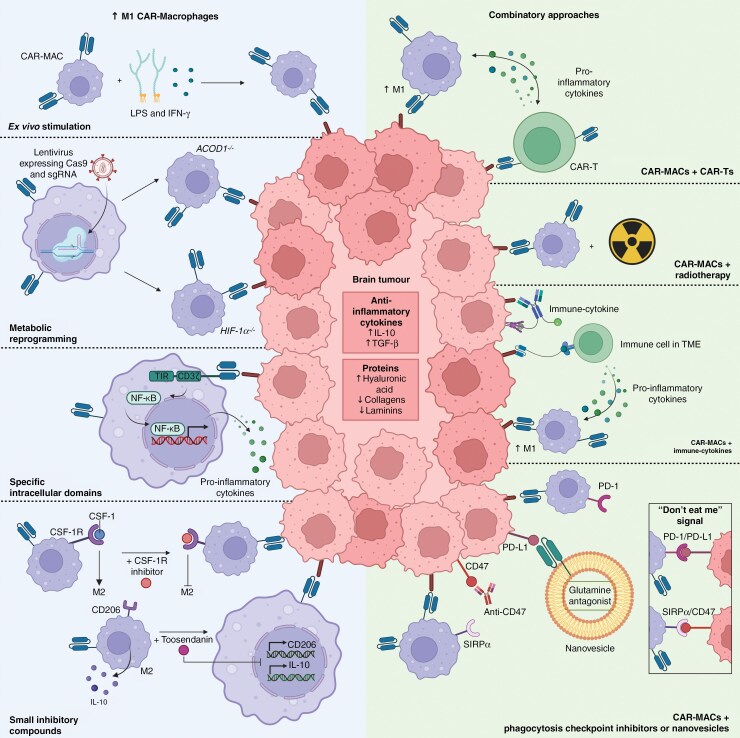
Chimeric antigen receptor (CAR)-macrophage applications for brain tumor treatment. Strategies to enhance M1 polarization of the CAR-MACs and combinatory approaches for higher efficacy targeting brain tumors.

CAR-MAC polarization towards the M1 phenotype seems favorable for enhanced antitumor activity and crucial to resist anti-inflammatory and inhibitory signals from the TME (Figure 3). One potential method to achieve this is by ex vivo activating the CAR-MACs with lipopolysaccharides (LPS) and Th1 cytokines. Bone-marrow-derived MACs (BMDMs) expressing first-generation anti-HER2 CARs enhanced CAR-MAC-mediated tumor cell phagocytosis and pro-inflammatory cytokine secretion following stimulation with LPS and recombinant mouse IFNγ.^74^ Intravenous infusion of the previously mentioned M1 polarized CAR-MACs in an ovarian cancer model, a lung metastasis model, and a subcutaneous melanoma model led to enhanced suppression of tumor growth compared to unstimulated CAR-MACs.

A second approach involves metabolic reprogramming of the MACs to prevent polarization towards the M2 phenotype. Recently, it was demonstrated that the anti-inflammatory system of MACs can be inhibited by deletion of the gene encoding aconitate decarboxylase (ACOD1).^82^ In this study a CRISPR/Cas9-mediated *ACOD1* knock-out in human induced pluripotent stem cells (iPSCs) urged the differentiation of the stem cells into MACs (iMACs). Subsequently, CAR molecules were transduced into these iMACs, resulting in *ACOD1*^-/-^CAR-iMACs. ACOD1-depletion effectively facilitated CAR-iMAC polarization towards the M1 phenotype, resulting in enhanced antitumor activities both in vitro and in vivo including tumor suppression and stronger phagocytosis compared to CAR-iMACs with intact ACOD1. Similarly, downregulating HIF-1α may enhance the antitumor activity of CAR-MACs, as HIF-1α-mediated epigenetic repression with CRIPR/Cas9 leads to MACs that predominantly exhibit an M1 phenotype with an antitumor function in hypoxic TME.^83^

Another strategy to promote M1 polarization in CAR-MACs involves (inhibitory) compounds to prevent M2 polarization. In vivo, research with a glioblastoma mouse model demonstrated that CSF-1R blocking effectively promoted M1 polarization of MACs and suppressed glioma growth.^45^ Similarly, the inhibitory small molecule toosendanin was shown to induce M1 polarization in a glioblastoma mouse model.^84^ These findings suggest that similar mechanisms could enhance the antitumor activity of CAR-MACs.

Alternatively, CAR-MACs with specific intracellular domains could be designed to enhance polarization towards an M1 phenotype after antigen binding, which was demonstrated by Lei et al.^77^ In this study, anti-EGFRvIII CAR molecules incorporated CD3ζ as an activation domain in tandem with the intracellular Toll/IL-1R (TIR) domain from toll-like receptor 4 as co-stimulatory domain. This configuration aims to enhance polarization towards the M1 phenotype by facilitating nuclear factor (NF)-κB/65 translocation into the nucleus to promote pro-inflammatory cytokine expression. The anti-EGFRvIII-CD3ζ-TIR-CAR-iMACs demonstrated alleviated tumor progression and prolonged survival in orthotopic glioblastoma mouse models with U87MG^EGFRvIII^ cells in comparison to first-generation anti-EGFRvIII-CAR-iMACs. Moreover, administration of the second-generation CARs resulted in elevated expression levels of M1 markers (eg, CD80) and elevated levels of pro-inflammatory cytokine TNF-α, known to induce apoptotis.^85^ These findings demonstrate that treatment with second-generation CAR-iMACs mediates antigen-dependent reduced brain tumor burden. In addition to the activation of an adaptive immune response and phagocytosis, CAR-iMACs can engage in efferocytosis to clear apoptotic cells. This unveils efferocytosis as an effector mechanism of CAR-iMACs in addition to phagocytosis and activation of an adaptive immune response.

### Combinatory CAR-MAC Approaches

As single therapies often fail to achieve optimal treatment outcomes for solid tumors, there is a growing interest in combination therapies to enhance the efficacy and safety of CAR therapy (Figure 3). One approach is to combine cellular immunotherapies for synergistic antitumor effects. Treatment of solid tumors with both CAR-MACs and CAR-T-cells led to an augmented killing effect and antitumor response in vitro compared to single-cell therapy treatment.^86^ Pro-inflammatory cytokines secreted by antigen-bound CAR-T-cells stimulate macrophage polarization towards an M1 phenotype, enhancing the antitumor response and cell-killing activity of CAR-MACs. Additionally, preliminary data shows that CAR-T-cells stimulate the recruitment of macrophage populations into the tumor microenvironment, amplifying macrophage-mediated antitumor effects.^87^ Designing a combined cell therapy with CAR-MACs and CAR-T-cells directed against distinct antigens could synergistically enhance the antitumor effect in brain tumors. This approach may also mitigate the risk of brain tumors evading treatment through antigen escape. The implementation of a combinatorial cell therapy targeting 2 distinct tumor-associated antigens is currently limited by restricted availability and knowledge of tumor-specific antigens. Therefore, prior to the actualization of such an approach for improved tumor killing, additional research into tumor-specific antigens is imperative.

A second approach for enhanced efficacy of CAR-MAC therapy is a combinatory approach with antibody-based immunotherapy utilizing phagocytosis checkpoint inhibitors. Phagocytosis checkpoints such as CD47, PD-L1, and CD24 are highly expressed on cancerous cells to evade macrophage clearance.^88^ Among the phagocytosis checkpoints expressed on tumor cells, CD47 has been most extensively researched. Inhibition of the CD47/signal regulatory protein α (CD47/SIRPα) pathway with antibodies enhances the antitumor activity of MACs.^89^ This was demonstrated in a study involving CAR-iMACs and anti-CD47, wherein a combined therapy exhibited enhanced antitumor activity in comparison to either anti-CD47 alone or anti-CD47 with a CAR-iMAC lacking intracellular activation domains, achieving nearly complete tumor signal eradication within 30 days in a HepG2 hepatocellular carcinoma mouse model.^77^ Similarly, the addition of anti-PD1 to CAR-MAC therapy has been shown to significantly reduce tumor volume in both orthotopic breast cancer 4T1-cHER2 and subcutaneous colorectal cancer CT26-hHER2 mouse models.^90^ These findings highlight the potential of synergistically combining CAR-MACs and phagocytosis checkpoint antibodies. Moreover, CAR-MAC therapy could be integrated with immune-cytokines, like their use in CAR-T-cell therapy, suppressing the TME or inducing macrophage polarization towards the M1 phenotype.

A novel approach to combinatory therapy involves the utilization of vesicles to metabolically undermine the immunosuppressive TME, thus enhancing CAR therapy. Recently, a nanovesicle was engineered with anti-PD-L1 scFvs on its surface, enabling binding to programmed death-ligand (PD-L1) expressed on tumor cells.^91^ Once bound, the nanovesicle delivers a glutamine antagonist, inhibiting tumor growth. This dual action of PD-L1 binding and glutamine antagonist release effectively reduces immunosuppressive cell presence and stimulates pro-inflammatory cytokine secretion. Consequently, the immunosuppressive TME is attenuated, leading to increased efficacy of CAR-T-cell therapy.^91^ This innovative approach holds promise for potentially enhancing the effectiveness of CAR-MAC therapy in a similar manner in brain tumors.

It is essential to realize that limited CAR-T therapy efficacy in solid tumors cannot solely be attributed to the TME, but also to antigen escape. To reduce the risk of antigen escape, radiotherapy is employed in CAR-T-cell therapy to enhance CAR target expression on tumor cells, induce immunogenic cell death and reshape the TME for immune response modulation, and increase antigen presentation by antigen-presenting cells.^92^ Murty et al. demonstrated that CAR-T-cells can induce tumor regression in syngeneic orthotopic mouse models of glioblastoma when combined with localized radiation.^93^ Radiotherapy could have the same role in enhancing CAR-MAC treatment, as radiotherapy at a 10 Gy dose significantly increases macrophage infiltration in tumors by 4.5 times, while reducing the M2 macrophage population by over 3.5 times.^94^ This shift favors a higher population of M1 MACs, which, in combination with CAR-MACs, could enhance antitumor activity. Moreover, radiotherapy combined with CD47 blockade induces a macrophage-mediated abscopal effect, where MACs enhance tumor clearance both at the irradiated site and in target distant, non-irradiated tumors.^70^ This highlights the potential for radiotherapy, alongside CD47 inhibition, to further boost the antitumor efficacy of CAR-MAC therapies. While radiation can improve CAR therapy by modifying the TME, it also introduces risks like immunosuppression, lymphocyte depletion, and increased toxicity.^95^ Therefore, careful evaluation is necessary to balance these benefits and risks. Given the complexity of brain tumors, with their heterogeneity and intricate TME, investigating various combinatory approaches through clinical trials is essential to enhance CAR-MAC therapy.

## Towards Clinical Translation

**Figure 4.**
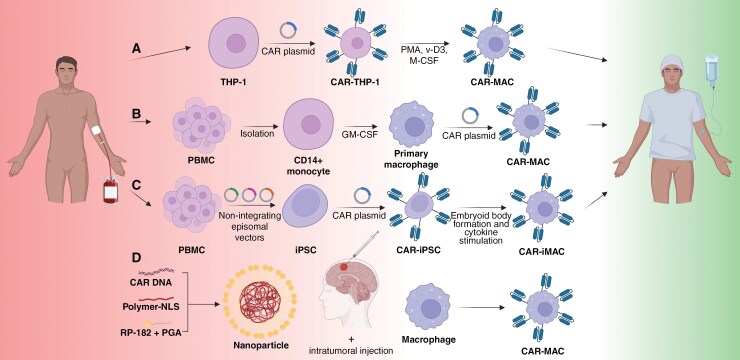
Generation of CAR-macrophages from various sources. Ex vivo manufacturing sources include (A) THP-1 cell line, (B) PBMCs or (C) iPSCs. (D) In vivo manufacturing uses primary macrophages as source. Abbreviations: CAR, chimeric antigen receptor; PMA, phorbol-12-myristate-13-acetate; v-D3, vitamin D3; M-CSF, macrophage colony-stimulating factor; PBMC, peripheral blood mononuclear cell; GM-CSF, granulocyte macrophage colony-stimulating factor; iPSC, induced pluripotent stem cell.

To achieve the desired therapeutic effect of cell-based therapy, one of the necessitates is the availability of high numbers of CAR-positive cells in the tumor. This is a significant challenge in CAR-T-cell therapy, primarily due to the constraints imposed by alloreactivity on the use of allogeneic cells. However, in CAR-MAC therapy, this challenge may arise if primary MACs were the sole macrophage source, particularly as MACs contain a broad and sensitive array of detectors of exogenous DNA, complicating transfection, and transduction.^96^ Fortunately, the macrophage source is not limited to primary cells; other sources include cell lines, peripheral blood mononuclear cells (PBMCs), and iPSCs (Figure 4a-c). The human macrophage cell line THP-1 was used for the first CAR-MACs with promising in vivo results.^69^ THP-1 is a leukemia monocytic cell line that can differentiate into THP-1-like MACs, following stimulation with phorbol-12-myristate-13-acetate (PMA), 25-dihydroxy vitamin D3 (v-D3) and macrophage colony-stimulating factor (M-CSF).^97^ THP-1-like MACs lack secretion of the pleiotropic cytokine IL-6 and the anti-inflammatory cytokine IL-10, thereby diminishing the likelihood of differentiation of neighboring MACs towards the M2 phenotype.^98^ This distinguishes them from PBMC-derived MACs, which exhibit more robust inflammatory characteristics.^97^ Despite PBMCs’ accessibility, their feasibility in clinical settings is limited due to inefficient genetic engineering.^97^ This positions THP-1 cells as a superior source for MACs biased towards M1 polarization. Nonetheless, the extent to which these THP-1-like MACs resemble primary MACs remains unclear. It is important to note that the expansion of MACs from these sources poses a significant challenge, representing a major barrier to the widespread application and development of CAR-MACs.^97^ Hence, sources of CAR-MACs have become a topic of increasing interest in recent years, with ‘off-the-shelf’ immunotherapy and in vivo manufacturing emerging with promising unlimited sources.

One of the sources for large-scale CAR-MAC production are iPSCs, which can be generated by stimulation of PBMCs using non-integrating episomal vectors. The engineering of iPSCs to express CAR molecules using viral vectors led to the generation of CAR-iPSCs. The CAR-iPSCs differentiated to CAR-iMACs through embryoid body formation and exogenous cytokine stimulation.^77,82,99^ CAR-iMACs showed antigen-dependent tumor cell phagocytosis and triggered the adaptive immune system (eg, T-cell activation), proving iPSCs as functional macrophage sources. Cell culture in stirred-tank bioreactors enables the mass production of iPSCs, overcoming the limitations of cell shortages for CAR-MAC therapy.^100^ However, concerns persist regarding the potential for tumorigenesis and immunogenicity associated with iPSCs as macrophage sources due to viral-vector engineering.

The ex vivo production of CAR-MACs from cell lines (THP-1), PBMCs, and iPSCs is a costly and intricate process.^97^ Therefore, the clinical application could be improved by in vivo production of CAR-MACs using non-viral gene delivery. By employing biodegradable poly (β-amino ester)-based nanoparticles decorated with macrophage-targeting and phenotype-switching RP-182 peptides, it becomes feasible to deliver DNA encoding a CAR to M2 TAMs in the TME, thereby inducing the generation of CAR-MACs displaying an antitumor M1 phenotype (Figure 4D).^79^ The in vivo generated CAR-MACs are proved successful in suppressing patient-derived brainstem glioma growth in a xenograft mouse model. Furthermore, CAR-MACs can be generated in vivo utilizing a brain extracellular matrix-mimetic hydrogel that incorporates CA-dextran-coated nanoporters.^78^ These nanoporters are released from the hydrogel in the resected tumor cavity, where they deliver plasmid DNA encoding a CAR to M2 TAMs. In a syngeneic orthotopic mouse glioma model, these CAR-MACs effectively suppressed tumor growth, with a combinatory approach with CD47 blockade further enhancing their therapeutic efficacy.^78^ While these innovative approaches remain in the preclinical phase, advancing them to clinical trials is essential. The first clinical trials using nanotechnology to generate CAR-bearing immune cells in vivo are either planned or already in progress.^101^ These approaches avoid viral-vector gene delivery, thereby mitigating the risk of tumorigenesis associated with viral gene integration. Consequently, in vivo production with M2 TAMs as a source emerges as a promising alternative to the costly and complicated ex vivo production process of CAR-MACs to combat brain tumors.

## Limitations

Research on, and the clinical implementation of CAR-MACs is in an emerging early stage, with data on clinical trial results of solid tumor treatment yet to be reported. Therefore, in vivo limitations remain to be discovered. Further research and ongoing clinical trials of CAR-MACs in solid tumor treatment are essential to assess cytotoxicity and efficacy accurately. It is imaginable that the hypoxic immunosuppressive environment within the TME could still pose a challenge to the effectiveness of CAR-MACs. However, this applies to the current adoptive cell therapies in general. Furthermore, clinical trials may reveal a decline of CAR-MAC efficacy over time, as CAR-MACs that exhibit an M1 antitumor phenotype can polarize reversibly towards an anti-inflammatory M2 phenotype. This “phenotypic uncertainty” could possibly be addressed by the development of MACs with a differentiated state, like the *ACOD1*-depleted CAR-MACs, but these have yet to be tested in clinical trials. Studies on CAR-MAC phenotype persistence are limited, but recent in vitro findings suggest that CD3ζ-CAR-MACs show a reduced M1 polarization after 7 days of tumor exposure, while second-generation CAR-MACs with a specific intracellular activation domain (CD3ζ-TIR-CAR-MACs) showed enhanced M1 polarization.^77^ Additionally, these CD3ζ-TIR-CAR-MACs exhibit the strongest resistance to M2 conversion, maintaining low CD163 levels. These in vitro findings were further validated in an orthotopic glioblastoma mouse model.^77^ However, the in vivo results are based on a 48-hour observation period, and longer-term effects were not assessed. CAR-MAC persistence remains an open question, and it is unclear if long-term persistence is required. If the tumor is fully eradicated, natural CAR-MAC decay may be adequate, similar to what is observed in CD19 B-cell lymphomas.^102–104^ Further research is essential to gain deeper insights into CAR-MAC persistence and optimize strategies for maintaining their antitumor activity over time.

## Conclusion and outlook

CAR-MAC therapy is a promising approach to treat brain tumors. Compared to traditional CAR-T-cell therapy, CAR-MAC therapy has significant advantages. In vitro and in vivo studies taught us that CAR-MACs show consistent therapeutic promise, are characterized by high tumor infiltration, exhibit low auto-immune toxicity, and show high cytotoxicity against brain tumor cells. Compared to CAR-T therapy, CAR-MACs can more efficiently infiltrate the TME, allowing them to reach their target location unhindered by abundant cell-matrix proteins, immunosuppressive cells, and anti-inflammatory cytokines present. CAR-MACs effectively clear tumor cells through phagocytosis and efferocytosis. Moreover, antigen-bound CAR-MACs secrete pro-inflammatory cytokines, and thereby boost an adaptive immune response that promotes the differentiation of M2 TAMs towards an antitumor M1 phenotype, resulting in the recruitment of other immune cells eg, T cells. For clinical translation, iPSCs can serve as a valuable source for the ex-vivo off-the-shelf production of CAR-MACs, enabling large-scale manufacturing. Additionally, the in vivo generation of CAR-MACs using M2 TAMs as a resource emerges as a promising alternative to the ex vivo production process of CAR-MACs. Further clinical studies are awaited to evaluate the safety and potency of CAR-MACs in brain malignancies.

CAR-MAC therapy still necessitates the development of new therapeutic tools or combinatory approaches to achieve maximum efficacy. Potential combinatory approaches that should be further investigated include CAR-MAC therapy with CAR-T-cell therapy, phagocytosis checkpoint antibodies, immune cytokines, genetically programmable vesicles, and radiotherapy. Furthermore, incorporating hypoxia-sensing CARs or ICRs are exciting directions that attract attention to enhance the functionality of CAR-MACs. With continued innovation, CAR-MACs hold the potential to significantly improve outcomes for solid (brain) cancer patients and pave the way for the next generation of cellular immunotherapies.

### Search Strategy and Selection Criteria

Relevant preclinical studies were identified in PubMed and Embase with the search terms “Brain tumor/cancer” and specific terms for Mesh or title/abstract screening (eg, “Glioblast*,” “glioblastoma[tiab]’’ OR ‘’Midline Glioma*” in combination with “Chimeric antigen receptor,” “CAR[tiab],” “CAR*,” “Macrophage’’ or specific cell types such as “Monocyte,” “IPSC,” “Myeloid,” “Dendritic*,” and “Microglial.” Relevant clinical studies were identified by using these same search terms on ClinicalTrials.gov. No date restrictions were applied to search queries; the last search of both databases was done on October 28, 2024. Only articles in English were reviewed. Publications corresponding to trials included in this Review were identified via a search of National Clinical Trial numbers on PubMed. The final reference list for the clinical studies was based on all current trials of CAR-T and CAR-MAC immunotherapy for brain/solid tumors.

## Supplementary Material

Supplementary material is available online at *Neuro-Oncology Advances* (https://academic.oup.com/noa).

vdaf059_suppl_Supplementary_Materials
