# Supplementary material for: Chimeric antigen receptor-macrophages: Emerging next-generation cell therapy for brain cancer
Source: Neurooncol Adv. 2025 Mar 19;7(1):vdaf059. doi: 10.1093/noajnl/vdaf059 (PMC12080554; doi:10.1093/noajnl/vdaf059)
Supplement: vdaf059_suppl_Supplementary_Materials [file vdaf059_suppl_supplementary_materials.docx]

**Supplementary Figures**

**Table S1. Summary of clinical trials that utilize CAR-T cells to target brain tumours.**

This table lists clinical trials and their respective targets towards brain tumours. Each clinical trial is assigned a unique colour; a repeating colour indicates that the same CAR set up is being tested across multiple brain tumours.

| **Clinical trial** | **Target** | **Cancer** |
| --- | --- | --- |
| NCT02331693 | EGFR | Advanced Glioma |
| NCT03500991 | HER2 | Atypical Teratoid/Rhabdoid Tumour |
| NCT03638167 | EGFR806 | Atypical Teratoid/Rhabdoid Tumour |
| NCT04185038 | B7-H3 | Atypical Teratoid/Rhabdoid Tumour |
| NCT05835687 | B7-H3 | Atypical Teratoid/Rhabdoid Tumour |
| NCT01454596 | EGFRvIII | Brain Cancer |
| NCT02541370 | CD133 | Brain Tumour |
| NCT05298995 | GD2 | Brain Tumour Adult |
| NCT05298995 | GD2 | Brain Tumour, Pediatric |
| NCT02442297 | HER2 | Brain Tumour, Recurrent |
| NCT02442297 | HER2 | Brain Tumour, Refractory |
| NCT05835687 | B7-H3 | Central Nervous System Neoplasms |
| NCT04185038 | B7-H3 | Central Nervous System Tumour |
| NCT03500991 | HER2 | Central Nervous System Tumour, Pediatric |
| NCT03638167 | EGFR806 | Central Nervous System Tumour, Pediatric |
| NCT03500991 | HER2 | Choroid Plexus Carcinoma |
| NCT03638167 | EGFR806 | Choroid Plexus Carcinoma |
| NCT04185038 | B7-H3 | Choroid Plexus Carcinoma |
| NCT04099797 | GD2 with C7R gene | Diffuse Intrinsic Pontine Glioma |
| NCT04185038 | B7-H3 | Diffuse Intrinsic Pontine Glioma |
| NCT04196413 | GD2 | Diffuse Intrinsic Pontine Glioma |
| NCT05298995 | GD2 | Diffuse Intrinsic Pontine Glioma |
| NCT05768880 | Combinations of B7-H3, EGFR806, HER2 and IL13-Zetakine | Diffuse Intrinsic Pontine Glioma |
| NCT06221553 | B7-H3 with truncated IL-7Ra | Diffuse Intrinsic Pontine Glioma |
| NCT04185038 | B7-H3 | Diffuse Midline Glioma |
| NCT05298995 | GD2 | Diffuse Midline Glioma |
| NCT05768880 | Combinations of B7-H3, EGFR806, HER2 and IL13-Zetakine | Diffuse Midline Glioma |
| NCT05544526 | GD2 | Diffuse Midline Glioma, H3 K27M-Mutant |
| NCT05835687 | B7-H3 | Diffuse Midline Glioma, H3 K27M-Mutant |
| NCT04099797 | GD2 with C7R gene | Embryonal Tumour |
| NCT05298995 | GD2 | Embryonal Tumour |
| NCT04099797 | GD2 with C7R gene | Ependymal Tumour |
| NCT03500991 | HER2 | Ependymoma |
| NCT03638167 | EGFR806 | Ependymoma |
| NCT04185038 | B7-H3 | Ependymoma |
| NCT04661384 | IL13Rα2 | Ependymoma |
| NCT04903080 | HER2 | Ependymoma |
| NCT05835687 | B7-H3 | Ependymoma |
| NCT02575261 | EphA2 | EphA2 Positive Malignant Glioma |
| NCT03500991 | HER2 | Germ cell Tumour |
| NCT03638167 | EGFR806 | Germ cell Tumour |
| NCT04185038 | B7-H3 | Germ cell Tumour |
| NCT01454596 | EGFRvIII | Glioblastoma |
| NCT02664363 | EGFRvIII | Glioblastoma |
| NCT04270461/NCT05131763 | NKG2D | Glioblastoma |
| NCT04661384 | IL13Rα2 | Glioblastoma |
| NCT05063682 | EGFRvIII | Glioblastoma |
| NCT05353530 | CD70 with IL8R modification | Glioblastoma |
| NCT05660369 | EGFRvIII or EGFR | Glioblastoma |
| NCT05835687 | B7-H3 | Glioblastoma |
| NCT06186401 | 1) EGFRvIII; 2) EphA2/IL-13R | Glioblastoma |
| NCT01109095 | HER2 | Glioblastoma Multiforme |
| NCT05063682 | EGFRvIII | Glioblastoma Multiforme |
| NCT05353530 | CD70 with IL8R modification | Glioblastoma Multiforme |
| NCT05366179 | B7-H3 | Glioblastoma Multiforme |
| NCT05627323 | MMP2 | Glioblastoma Multiforme of Brain |
| NCT02844062 | EGFRvIII | Glioblastoma Multiforme, Recurrent |
| NCT05474378 | B7-H3 | Glioblastoma Multiforme, Recurrent |
| NCT05868083 | Personalized with EGFR, EGFRvIII, HER2 or IL13Rα2 | Glioblastoma Multiforme, Recurrent |
| NCT06616727 | EGFR, EGFRvIII, HER2, IL13Rα2 | Glioblastoma Multiforme, Recurrent |
| NCT02208362 | IL13Rα2 with truncated CD19 | Glioblastoma, Recurrent |
| NCT03283631 | EGFRvIII | Glioblastoma, Recurrent |
| NCT04003649 | IL13Rα2 | Glioblastoma, Recurrent |
| NCT04045847 | CD147 | Glioblastoma, Recurrent |
| NCT04077866 | B7-H3 | Glioblastoma, Recurrent |
| NCT04214392 | MMP2 | Glioblastoma, Recurrent |
| NCT04385173 | B7-H3 | Glioblastoma, Recurrent |
| NCT04717999 | NKG2D | Glioblastoma, Recurrent |
| NCT05241392 | B7-H3 | Glioblastoma, Recurrent |
| NCT05577091 | CD44 and CD133 with truncated IL7Ra | Glioblastoma, Recurrent |
| NCT05660369 | EGFRvIII or EGFR | Glioblastoma, Recurrent |
| NCT05802693 | EGFRvIII | Glioblastoma, Recurrent |
| NCT06186401 | 1) EGFRvIII; 2) EphA2/IL-13R | Glioblastoma, Recurrent |
| NCT02208362 | IL13Rα2 with truncated CD19 | Glioblastoma, Refractory |
| NCT04003649 | IL13Rα2 | Glioblastoma, Refractory |
| NCT04077866 | B7-H3 | Glioblastoma, Refractory |
| NCT04385173 | B7-H3 | Glioblastoma, Refractory |
| NCT03283631 | EGFRvIII | Gliocarsoma, Recurrent |
| NCT03423992 | Personalized with EGFRvIII, IL13Rα2, HER-2, CD133 or GD2 | Glioma |
| NCT03500991 | HER2 | Glioma |
| NCT03638167 | EGFR806 | Glioma |
| NCT04185038 | B7-H3 | Glioma |
| NCT04406610 | GD2 | Glioma of Brain |
| NCT02208362 | IL13Rα2 with truncated CD19 | Glioma WHO grade II and III, Recurrent |
| NCT04214392 | MMP2 | Glioma WHO grade II and III, Recurrent |
| NCT02208362 | IL13Rα2 with truncated CD19 | Glioma WHO grade II and III, Refractory |
| NCT05063682 | EGFRvIII | Glioma, Malignant |
| NCT05660369 | EGFRvIII or EGFR | Glioma, Recurrent |
| NCT01454596 | EGFRvIII | Gliosarcoma |
| NCT02664363 | EGFRvIII | Gliosarcoma |
| NCT04099797 | GD2 with C7R gene | High Grade Glioma |
| NCT05298995 | GD2 | High Grade Glioma |
| NCT05835687 | B7-H3 | High Grade Glioma |
| NCT06482905 | B7-H3 | High Grade Glioma |
| NCT04510051 | IL13Rα2 | Malignant Brain Neoplasm |
| NCT04510051 | IL13Rα2 | Malignant Brain Neoplasm, Recurrent |
| NCT04510051 | IL13Rα2 | Malignant Brain Neoplasm, Refractory |
| NCT05660369 | EGFRvIII or EGFR | Malignant Glioma |
| NCT02617134 | MUC1 | Malignant Glioma of Brain |
| NCT03423992 | Personalized with EGFRvIII, IL13Rα2, HER-2, CD133 or GD2 | Malignant Glioma of Brain |
| NCT02208362 | IL13Rα2 with truncated CD19 | Malignant Glioma, Recurrent |
| NCT04045847 | CD147 | Malignant Glioma, Recurrent |
| NCT04214392 | MMP2 | Malignant Glioma, Recurrent |
| NCT02208362 | IL13Rα2 with truncated CD19 | Malignant Glioma, Refractory |
| NCT03500991 | HER2 | Medulloblastoma |
| NCT03638167 | EGFR806 | Medulloblastoma |
| NCT04270461/NCT05131763 | NKG2D | Medulloblastoma |
| NCT04661384 | IL13Rα2 | Medulloblastoma |
| NCT05835687 | B7-H3 | Medulloblastoma |
| NCT04185038 | B7-H3 | Medulloblastoma, Childhood |
| NCT05298995 | GD2 | Medulloblastoma, Childhood |
| NCT03696030 | HER2 | Metastatic Malignant Neoplasm in the Brain |
| NCT03696030 | HER2 | Metastatic Malignant Neoplasm in the Leptomeninges |
| NCT04661384 | IL13Rα2 | Metastatic Malignant Neoplasm in the Leptomeninges, Recurrent |
| NCT06186401 | 1) EGFRvIII; 2) EphA2/IL-13R | MGMT-Unmethylated Glioblastoma |
| NCT03500991 | HER2 | Pineoblastoma |
| NCT03638167 | EGFR806 | Pineoblastoma |
| NCT04185038 | B7-H3 | Pineoblastoma, Childhood |
| NCT05768880 | Combinations of B7-H3, EGFR806, HER2 and IL13-Zetakine | Primary Malignant Central Nervous System Neoplasm, Refractory |
| NCT03500991 | HER2 | Primitive Neuroectodermal Tumour |
| NCT03638167 | EGFR806 | Primitive Neuroectodermal Tumour |
| NCT04185038 | B7-H3 | Primitive Neuroectodermal Tumour |
| NCT03423992 | Personalized with EGFRvIII, IL13Rα2, HER-2, CD133 or GD2 | Recurrence Tumour |
| NCT05540873 | IL13Rα2 | Recurrent Malignant Glioma |
| NCT05768880 | Combinations of B7-H3, EGFR806, HER2 and IL13-Zetakine | Recurrent CNS Tumour, Adult |
| NCT05768880 | Combinations of B7-H3, EGFR806, HER2 and IL13-Zetakine | Recurrent CNS Tumour, Childhood |
| NCT04196413 | GD2 | Spinal Diffuse Midline |

Abbreviatons: EGFR; Epidermal Growth Factor Receptor, HER2; Human Epithelial Growth Factor Receptor 2, IL 7Rα; Interleukin 7 Receptor a, IL 8; Interleukin 8, IL 13; Interleukin 13, EphA2; Ephrin Receptor A2, MMP2, Metalloproteinase 2, MUC1; Mucin 1

**Table S2. Current In vivo studies that use CAR-MACs.** This table lists the respective targets and activation domains of the CAR-MACs, along with their cell source, in vivo model, delivery route, primary endpoint, and their reference.

| **Target** | **Activation domain** | **Cell source** | ***In vivo*** | **Delivery route** | **Primary endpoint** | **Reference** |
| --- | --- | --- | --- | --- | --- | --- |
| CSPG4 | FcRγ | Bone marrow-derive macrophages | Xenograft mouse model of human metastatic melanoma cell line A375 in NRG mice via subcutaneous graft | Peritumour | Tumour volume | Greiner^1^ |
| HER2 | CD3ζ | THP-1 | Xenograft mouse model of human SKOV3 ovarian cancer cells in NSGS mice;  Humanized immune system mouse model of SKOV3 ovarian cancer cells in NSG mice. | Intravenous; Intraperitoneal | Tumour burden, survival, | Klichinski^2^ |
| PD-L1 | CD3ζ | Lin^-^ hematopoietic stem cells | Syngeneic model of BALB/c mice via subcutaneous graft of 4T1 triple negative breast cancer cells | Intravenous | Tumour volume, mouse body weight, survival | Myers Chen^3^ |
| CD19 | CD3ζ | Human pluripotent stem cells | Xenograft mouse model of human Burkitt’s lymphoma Raji cells in NOG mice via intraperitoneal injection;  Xenograft mouse model of human breast cancer MDA-MB-231 cells in NOG mice via intraperitoneal injection | Intraperitoneal | Tumour burden, survival, tumour volume | Shen^4^ |
| PSCA | CD3ζ | Human induced pluripotent stem cells from hematopoietic stem and progenitor cells | Xenograft mouse model of human pancreatic cancer Capan-1 in NSG-SGM3 mice via intraperitoneal injection | Intraperitoneal; Intravenous | Tumour burden, survival | Shah^5^ |
| HER2 | CD3ζ-CD147 | THP-1 | Xenograft mouse model of human SKOV3 ovarian cancer cells or NCI-N87 gastric cancer cells in BALB/c nude mice via subcutaneous graft;  Xenograft mouse model of human MDA-MB-453 breast cancer cells in BALB/c nude mice via orthotopic transplantation;  Xenograft mouse model of human SKOV3 ovarian cancer in NSG mice via subcutaneous graft | Intravenous | Tumour volume, tumour weight | Yang^6^ |
| HER2 / CD47 | 4-1BB-CD3ζ | THP-1 | Syngeneic model of NCG mice with Hu-PBMC via subcutaneous graft of SKOV3 ovarian cancer cells  Xenograft mouse model of human SKOV3 ovarian cancer cells in BALB/c nude mice via subcutaneous graft | Intrathecal | Tumour volume, tumour weight, tumorigenicity | Chen^7^ |
| HER2 | FcεR1γ | Human peritoneal macrophages | Xenograft mouse model of human MKN45 gastric cancer cells in BALB/c nude mice via intraperitoneal injection | Intraperitoneal | Tumour burden, tumour weight (diameter >3 mm) or tumour nodules count (diameter <3 mm) | Dong^8^ |
| VEGFR2 | Tlr4-Ifngr1-Ifngr2, Tlr4, Ifngr1-Ifngr1 | RAW264.7 cell line | Syngeneic model of BALB/c mice via subcutaneous graft of 4T1 triple negative breast cancer cells | Intravenous | Tumour volume, tumour weight | Duan^9^ |
| GD2 | OX40-CD3ζ | Human pluripotent stem cells | Xenograft mouse model of human CHLA-20 neuroblastoma cells in NSG mice via subcutaneous graft | Subcutaneous | Tumour burden, mouse body weight | Zhang^10^ |
| HER2 | FcγRI | Bone marrow-derived macrophages and primary macrophages | Syngeneic mouse model of C57BL/6 mice via intraperitoneal injection of ID8 ovarian surface epithelial cells or via subcutaneous graft and intravenous injection of B16F10 melanoma cells | Intraperitoneal;  Intravenous | Tumour burden, survival, tumour volume, mouse body weight | Huo^11^ |
| CCR7 | MerTK, 4-1BB-CD3ζ | RAW264.7 cell line | Syngeneic mouse model of BALB/c mice via subcutaneous graft of 4T1 triple negative breast cancer cells | Intravenous | Tumour volume, survival, mouse body weight, mouse temperature | Niu^12^ |

Abbreviations: CSPG4: Chondroitin sulfate proteoglycan 4, HER2; Human Epithelial Growth Factor Receptor 2, PD-L1; Programmed Death Receptor Ligand 1, PSCA; Prostate Stem Cell Antigen, VEGFR2; Vascular Endothelial Growth Factor Receptor 2, CCR7; C-C Chemokine Receptor 7

**References**

1. Greiner D, Xue Q, Waddell TQ, et al. CSPG4-targeting CAR-macrophages inhibit melanoma growth [Preprint]. *bioRxiv*. Published online 2024. doi:10.1101/2024.06.04.597413

2. Klichinsky M, Ruella M, Shestova O, et al. Human chimeric antigen receptor macrophages for cancer immunotherapy. *Nat Biotechnol*. 2020;38(8):947-953. doi:10.1038/s41587-020-0462-y

3. Myers Chen K, Grun D, Gautier B, et al. Targeting PD-L1 in solid cancer with myeloid cells expressing a CAR-like immune receptor. *Front Immunol*. 2024;15. doi:10.3389/fimmu.2024.1380065

4. Shen J, Lyu S, Xu Y, et al. Activating innate immune responses repolarizes hPSC-derived CAR macrophages to improve anti-tumor activity. *Cell Stem Cell*. 2024;31(7):1003-1019.e9. doi:10.1016/j.stem.2024.04.012

5. Shah Z, Tian L, Li Z, et al. Human anti-PSCA CAR macrophages possess potent antitumor activity against pancreatic cancer. *Cell Stem Cell*. 2024;31(6):803-817.e6. doi:10.1016/j.stem.2024.03.018

6. Yang B, Wang X, Wei X, Ma J. Development of a novel HER2-CAR monocyte cell therapy with controllable proliferation and enhanced anti-tumor efficacy. *Chin Med J (Engl)*. Published online January 19, 2024. doi:10.1097/CM9.0000000000002944

7. Chen Y, Zhu X, Liu H, et al. The application of HER2 and CD47 CAR-macrophage in ovarian cancer. *J Transl Med*. 2023;21(1):654. doi:10.1186/s12967-023-04479-8

8. Dong X, Fan J, Xie W, et al. Efficacy evaluation of chimeric antigen receptor-modified human peritoneal macrophages in the treatment of gastric cancer. *Br J Cancer*. 2023;129(3):551-562. doi:10.1038/s41416-023-02319-6

9. Duan Z, Li Z, Wang Z, Chen C, Luo Y. Chimeric antigen receptor macrophages activated through TLR4 or IFN-γ receptors suppress breast cancer growth by targeting VEGFR2. *Cancer Immunology, Immunotherapy*. 2023;72(10):3243-3257. doi:10.1007/s00262-023-03490-8

10. Zhang J, Webster S, Duffin B, et al. Generation of anti-GD2 CAR macrophages from human pluripotent stem cells for cancer immunotherapies. *Stem Cell Reports*. 2023;18(2):585-596. doi:10.1016/j.stemcr.2022.12.012

11. Huo Y, Zhang H, Sa L, et al. M1 polarization enhances the antitumor activity of chimeric antigen receptor macrophages in solid tumors. *J Transl Med*. 2023;21(1):1-15. doi:10.1186/s12967-023-04061-2

12. Niu Z, Chen G, Chang W, et al. Chimeric antigen receptor‐modified macrophages trigger systemic anti‐tumour immunity. *J Pathol*. 2021;253(3):247-257. doi:10.1002/path.5585
